# Theoretical DFT Analysis of a Polyacrylamide/Amylose Copolymer for the Removal of Cd(II), Hg(II), and Pb(II) from Aqueous Solutions

**DOI:** 10.3390/polym17141943

**Published:** 2025-07-16

**Authors:** Joaquin Hernandez-Fernandez, Yuly Maldonado-Morales, Rafael Gonzalez-Cuello, Ángel Villabona-Ortíz, Rodrigo Ortega-Toro

**Affiliations:** 1 Chemistry Program, Department of Natural and Exact Sciences, San Pablo Campus, University of Cartagena, Cartagena 130015, Colombia; ymaldonadom@unicartagena.edu.co; 2 Chemical Engineering Program, School of Engineering, Universidad Tecnológica de Bolívar, Parque Industrial y Tecnológico Carlos Vélez Pombo, Cartagena 130001, Colombia; 3 Department of Natural and Exact Science, Universidad de la Costa, Barranquilla 080002, Colombia; 4 Food Packaging and Shelf-Life Research Group (FP&SL), Food Engineering Program, University of Cartagena, Cartagena 130015, Colombia; rgonzalezc1@unicartagena.edu.co (R.G.-C.); rortegap1@unicartagena.edu.co (R.O.-T.); 5 Process Design and Biomass Utilization Research Group (IDAB), Department of Chemical Engineering, Universidad de Cartagena, Avenida del Consulado St. 30, Cartagena 130015, Colombia; avillabonao@unicartagena.edu.co

**Keywords:** heavy metals, acrylamide, amylose, copolymers, DFT, adsorption, water treatment

## Abstract

This study theoretically investigates the potential of a polyacrylamide copolymerized with amylose, a primary component of starch, to evaluate its efficiency in removing heavy metals from industrial wastewater. This material concept seeks to combine the high adsorption capacity of polyacrylamide with the low cost and biodegradability of starch, ultimately aiming to offer an economical, efficient, and sustainable alternative for wastewater treatment. To this end, a computational model based on density functional theory (DFT) was developed, utilizing the B3LYP functional with the 6-311++G(d,p) basis set, a widely recognized combination that strikes a balance between accuracy and computational cost. The interactions between an acrylamide-amylose (AM/Amy) polymer matrix, as well as the individual polymers (AM and Amy), and the metal ions Pb, Hg, and Cd in their hexahydrated form (M·6H_2_O) were analyzed. This modeling approach, where M represents any of these metals, simulates a realistic aqueous environment around the metal ion. Molecular geometries were optimized, and key parameters such as total energy, dipole moment, frontier molecular orbital (HOMO-LUMO) energy levels, and Density of States (DOS) graphs were calculated to characterize the stability and electronic reactivity of the molecules. The results indicate that this proposed copolymer, through its favorable electronic properties, exhibits a high adsorption capacity for metal ions such as Pb and Cd, positioning it as a promising material for environmental applications.

## 1. Introduction

Heavy metals stand as priority pollutants among the vast array of toxic substances released into water, largely because of their significant bioaccumulation capacity and persistence within the environment [1]. These hazardous elements originate from diverse industrial, agricultural, mining, and pyrometallurgical activities [1]. Among the most dangerous and frequently discarded by these sectors are (Cd), chromium (Cr), copper (Cu), nickel (Ni), arsenic (As), lead (Pb), and zinc (Zn) [2,3].

Due to their high solubility in aquatic environments, heavy metals are commonly present in dissolved ionic forms, which facilitates their absorption by living organisms. Once introduced into the food chain, these metals can accumulate in human tissues, posing a serious health risk if their concentrations exceed permissible limits [4]. Cadmium, mercury, and lead are particularly noteworthy for their high toxicity. Cadmium, for instance, is dangerous even in minute quantities, with adverse effects including muscle degeneration, osteoporosis, hearing loss, and damage to the cardiovascular and nervous systems [5]. Mercury, a potent neurotoxin, impacts the central nervous system and has been linked to neurodegenerative diseases, developmental disorders, cancer, and reproductive dysfunction [6]. Similarly, lead negatively affects the endocrine system and kidneys, besides impairing reproductive function, and its excessive accumulation in the body can unfortunately be lethal [7].

In light of these risks, it is crucial to remove heavy metals from aquatic ecosystems to mitigate their impact on the environment and human health. There are multiple methods for removing contaminating metals in aqueous environments; among the most widely used are ion exchange [8], membrane filtration [9], electrochemical treatment [10], chemical precipitation [11,12], and adsorption [13]. Among these, adsorption is considered the most efficient and non-destructive method for removing dyes and metal ions from aqueous solutions [14]. This process fundamentally involves a mass transfer phenomenon, wherein a substance transitions from a liquid phase and becomes fixed onto the surface of a solid material through either physical or chemical interactions [15].

Indeed, the removal of metal ions from industrial effluents represents one of adsorption’s most significant applications. This method is particularly notable for its versatility in the synthesis and modification of adsorbent materials, many of which can be regenerated for multiple uses [16]. The development of various adsorbent materials, such as activated carbon [17], chitosan [18], biomass [19], and a range of natural and synthetic polymers [20], has solidified adsorption as one of the most widely adopted techniques in water treatment in recent years [21].

Within this expanding field, hydrogels have emerged as a highly efficient alternative for removing heavy metals via adsorption. Their appeal stems from their unique three-dimensional cross-linked structure and exceptional capacity for water retention [12]. These highly porous materials exhibit viscoelastic properties, biocompatibility, and impressive absorption capabilities [22,23]. Moreover, their hydrophilicity, mechanical strength, and self-sustainability have led to extensive research into their use as adsorbent membranes [24], positioning them as a promising avenue for developing novel technologies to treat heavy metal-contaminated water.

Beyond their broad utility in various fields, including controlled drug delivery systems [25], wound dressings [26], tissue engineering [27,28,29], and sensors [30], hydrogels are uniquely effective for heavy metal removal [12]. Their inherent ability to adsorb heavy metals is primarily attributed to their favorable surface chemistry and the presence of numerous hydrophilic functional groups, such as carboxyl (-COOH), amino (-NH_2_), hydroxyl (-OH), and sulfonyl (-SO_3_H), which readily facilitate interactions with metal ions [31].

To achieve these desirable properties, hydrogels are frequently synthesized using hydrophilic polymers. Notable examples include polyethylene glycol (PEG) [32], polyvinyl alcohol (PVA) [33], gelatin [34], and polyacrylamide (PAM) [34]. Additionally, various carbohydrate polymers like chitosan (CS) [35], hyaluronic acid (HA) [36], carboxymethylcellulose (CMC) [37], sodium alginate [38], and starch [39] are commonly employed. Polyacrylamide, a polymer formed by the polymerization of acrylamide monomers, which, interestingly, can also be generated naturally in some starchy foods cooked at high temperatures [40], is broadly utilized across industries. Under normal conditions, synthetic PAM is a neutral compound, but its properties can be modified significantly; when copolymerized with other monomers, it can acquire anionic or cationic charges [41]. PAM’s high water retention capacity, hydrophilic nature, and ease of processing make it particularly valuable in fields such as the pharmaceutical industry [42]. Recent studies have indeed demonstrated that polyacrylamide, when modified with polymeric materials containing specific functional groups, is highly effective for removing metals from aqueous solutions [43]. Among these modifying polymers, starch stands out as a natural polysaccharide, itself composed of amylose and amylopectin [44]. A higher proportion of amylose within starch consistently promotes the formation of hydrogels with highly porous structures and enhanced capacities for both water and heavy metal adsorption, notably improving efficiency within a pH range of 4 to 4.5 [45].

Recent advancements in the field have seen the development of novel polymer-based composites and the increasing use of computational approaches to understand and optimize heavy metal remediation. For example, in 2019, polyacrylamide (PAM) and sodium montmorillonite (Na-MMT) nanocomposites were synthesized via free radical polymerization, achieving impressive removal efficiencies of 99.3% for Ni and 98.7% for Co at pH 6, effectively surpassing the performance of the individual materials [46]. That same year, new polyacrylamide nanocomposites grafted onto Cell@Fe_3_O_4_ were developed for Pb adsorption, with their variables—such as contact time, pH, and concentration—optimized using artificial neural networks (ANNs), leading to a maximum adsorption capacity of 314.47 mg/g at 323 K [47]. Also in 2019, density functional theory (DFT) was employed to investigate the adsorption of various heavy metal ions (Cd, Cr, Cu, Hg, Pb, and Zn) onto a methacrylate monomer derived from vanillin (VMA). This computational work revealed that interactions with nitrogen were stronger than those with oxygen, favoring the adsorption of Cu, Cr, and Pb due to their greater energy stability, and a Quantum Theory of Atoms in Molecules (QTAIM) analysis indicated a partially covalent interaction, positioning VMA as a promising candidate for contaminated water remediation [48]. More recently, in 2024, a predictive model integrating response surface methodology (RSM), artificial neural networks (ANNs), and machine learning (ML) was developed to optimize dye removal and Gibbs free energy in adsorption processes using a chitosan-polyacrylamide/TiO_2_ composite. This model demonstrated high accuracy in predicting adsorbent behavior, with an R^2^ value of 0.999, further solidifying the role of advanced modeling techniques in contaminant treatment [49].

This study theoretically investigates the potential of a novel polyacrylamide copolymerized with amylose, a primary component of starch, to evaluate its efficiency in removing heavy metals from aqueous solutions. This material concept seeks to combine the high adsorption capacity of polyacrylamide with the low cost and biodegradability of starch, ultimately aiming to offer an economical, efficient, and sustainable alternative for wastewater treatment. The novelty of this work lies in providing a fundamental, molecular-level understanding of the interactions between this specific hybrid copolymer (AM/Amy) and key heavy metal ions (Pb, Hg, and Cd) in their hexahydrated form (M·6H_2_O), a crucial aspect not extensively explored computationally. To this end, a computational model based on density functional theory (DFT) was developed, utilizing the B3LYP functional with the 6-311++G(d,p) basis set, a widely recognized combination that balances accuracy and computational cost. The interactions between the acrylamide-amylose (AM/Amy) copolymer, as well as its individual polymer segments (AM and Amy), and the target metal ions were meticulously analyzed. This modeling approach simulates a realistic aqueous environment around the metal ion, where M represents any of these metals. Molecular geometries were optimized, and key parameters such as total energy, dipole moment, frontier molecular orbital (HOMO-LUMO) energy levels, and Density of States (DOS) graphs were calculated to characterize the stability and electronic reactivity of the polymer fragments. The results indicate that this proposed copolymer fragment, through its favorable electronic properties, exhibits a high adsorption capacity for metal ions such as Pb, Hg, and Cd, positioning it as a promising material for environmental applications and providing critical insights for the rational design of next-generation adsorbents.

## 2. Materials and Methods

### Computational Details

In this study, density functional theory (DFT) calculations were employed to analyze the molecular-level interactions between the heavy metal cations cadmium (Cd), mercury (Hg), and lead (Pb), and a novel amylose-modified acrylamide copolymer. The primary objective was to computationally evaluate the intrinsic binding affinity and adsorption mechanism of this material for removing these metals, explicitly modeled in their hydrated form (M·6H_2_O). This analysis was crucial for understanding the synergistic effects observed in the copolymer’s performance compared to those of its individual polymer components, acrylamide (AM) and amylose (Amy), and for elucidating the underlying adsorption mechanisms.

All DFT calculations were performed using the GAUSSIAN 16 software package. To ensure an appropriate level of theory capable of accurately describing diverse interactions within our molecules (including covalent bonding, non-covalent interactions, and coordination), a preliminary method comparison was conducted. This assessment involved isolated monomer units of acrylamide (AM) and amylose (Amy), as well as heavy metal ions. We compared three widely used density functionals with their respective basis sets: the Becke three-parameter Lee-Yang-Parr B3LYP/6-311++G(d,p) [50], selected as a well-established standard for general chemical accuracy and its favorable computational cost; Truhlar’s M05-2X/6-31+G(d,p) [51], tested for its potential to provide sufficient accuracy for non-covalent interactions at a lower computational cost; and Truhlar’s M06-2X/6-311++G(d,p) [52], chosen for its demonstrated accuracy in describing non-covalent interactions, hydrogen bonding, and dispersion effects, which are critical for intermolecular interactions in adsorption processes. After this preliminary assessment, the B3LYP functional combined with the 6-311++G(d,p) basis set was ultimately selected for all subsequent calculations. This choice was based on its established reliability for modeling various chemical models, its favorable computational cost, and its consistent ability to identify the most thermodynamically stable (lowest energy) binding configurations for our foundational molecular fragments.

Molecular models of the acrylamide-amylose copolymer (AM/Amy) were designed, consisting of a 2AM:2Amy polymer segment. This fragment size, representing two acrylamide monomer units chemically linked to two amylose monomer units, was chosen to effectively capture the essential local bonding environment and functional groups relevant for adsorption while remaining computationally feasible. The interactions of these copolymer fragments, as well as the individual AM and Amy polymer segments, with the heavy metals were then systematically evaluated. To accurately simulate the metals’ most probable coordination environment when dissolved in water, the heavy metal ions were explicitly modeled as hexahydrated species (M·6H_2_O).

Each structure, including isolated polymer fragments, hydrated metal ions, and their complexes, underwent thorough geometry optimization to locate the local energy minimum on the potential energy surface. Following optimization, frequency calculations were performed to confirm that each optimized structure corresponded to a true energy minimum (i.e., no imaginary frequencies). From these optimized structures, several key electronic and structural parameters were calculated to analyze the interaction and reactivity. Frontier molecular orbital energies (HOMO-LUMO) were determined, as these provide insights into the electronic stability and chemical reactivity of the polymer fragments, indicating its propensity for electron donation (HOMO) or acceptance (LUMO). To further analyze the electronic contributions of individual fragments and their distribution across energy levels, Density of States (DOS) graphs [53] were generated using the MultiWFN 3.8 software package. The Total Dipole Moment (TDM) was also calculated, a parameter useful for evaluating the overall polarity of the structures and their potential for electrostatic interactions with the charged metal ions, influencing their orientation during adsorption. Finally, molecular electrostatic potential (MEP) maps were generated; these maps visually represent the charge distribution on the molecular surface, identifying potential nucleophilic (electron-rich) and electrophilic (electron-deficient) regions, thereby indicating possible adsorption sites for the metals. These comprehensive calculations allowed for a detailed analysis of the interaction mechanisms between each polymer matrix and the heavy metals, providing foundational insights into their adsorption capabilities.

## 3. Results

### 3.1. Choice of Method

The preliminary method comparison, summarized in Table 1, showed that the B3LYP functional consistently predicted lower energies for the optimized structures of the hydrated cadmium (Cd), lead (Pb), and mercury (Hg) metal complexes, as well as the acrylamide (AM) and amylose (Amy) polymer segments. These lower energies suggest greater thermodynamic stability for the complexes when modeled with B3LYP, indicating its effectiveness in identifying the most stable binding configurations. While the calculated entropy values across all three methods (B3LYP, M05-2X, and M06-2X) were of a similar order of magnitude, implying comparable vibrational descriptions, B3LYP ultimately achieved an overall superior balance between energy stability and entropic contributions. This comprehensive evaluation solidified B3LYP’s selection as the optimal functional for all subsequent calculations in this study.

As presented in Table 1, the B3LYP functional, combined with the 6-311++G(d,p) basis set, consistently yielded the lowest total energy values for all studied complexes, encompassing hydrated metal (M·6H_2_O) and individual polymer structures (AM and Amy). This consistent prediction of lower energies by B3LYP indicates greater thermodynamic stability for the modeled species.

Furthermore, the entropy values calculated with B3LYP were consistently in the same order of magnitude as those obtained from the M05-2X and M06-2X methods. For example, in the case of Cd·6H_2_O, the entropies were 73.214, 71.839, and 72.427 Cal/mol·K for B3LYP, M05-2X, and M06-2X, respectively. This quantitative similarity in vibrational descriptions across all methods implies that entropic contributions do not significantly differentiate the functionals, thereby allowing the total energy term to serve as the primary driver for stability comparisons. In this context, B3LYP demonstrated a superior balance between energy stability and entropic contributions by consistently providing lower total energies while maintaining comparable entropic effects, which resulted in more favorable overall Gibbs free energies.

Although the M05-2X and M06-2X functionals occasionally predicted higher dipole moments in some instances (Cd·6H_2_O and AM, where M05-2X values were 2.8165 and 4.2997 Debye, respectively, compared to B3LYP’s 1.7821 and 4.1389 Debye), which could theoretically favor interactions with polar media or charged surfaces, this advantage did not compensate for their generally higher total energies or the increased computational cost associated with these methods. While the Gibbs free energies obtained with the three functionals were generally comparable, B3LYP consistently provided slightly more negative or equivalent values, further reinforcing its suitability for accurately evaluating the stability and spontaneity of the investigated molecules.

**Figure 1:**
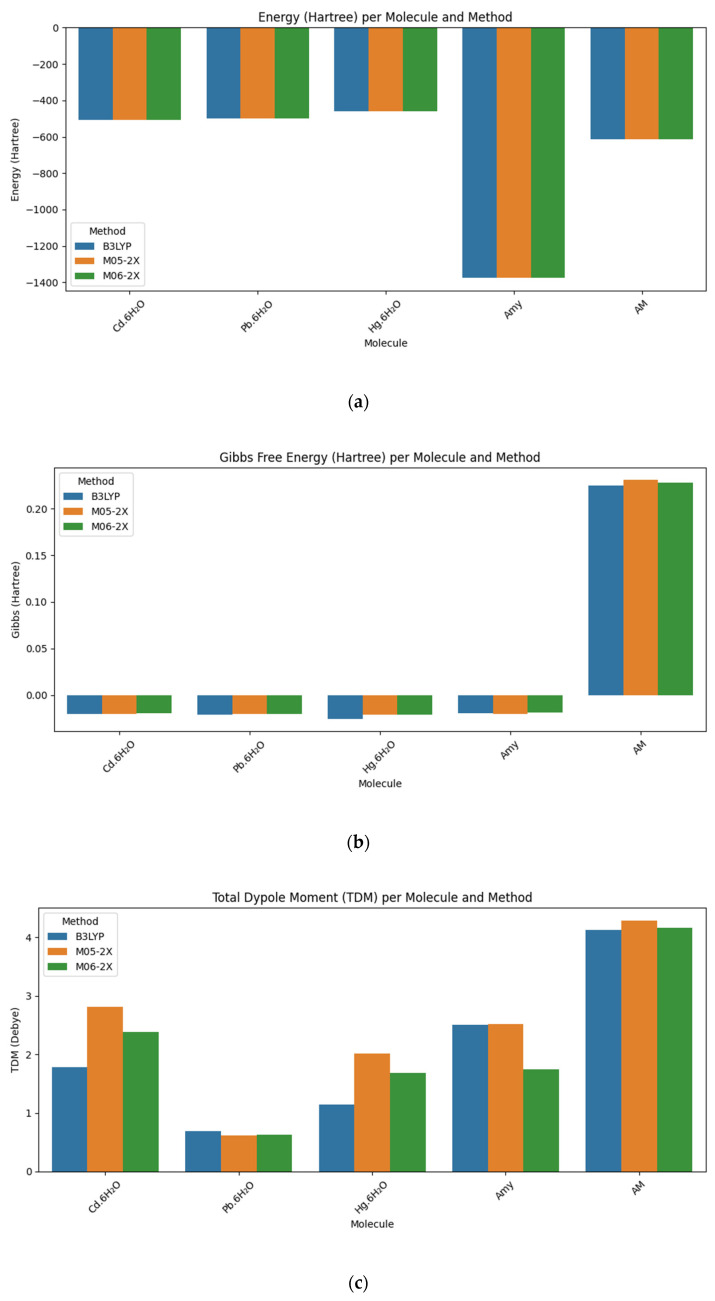

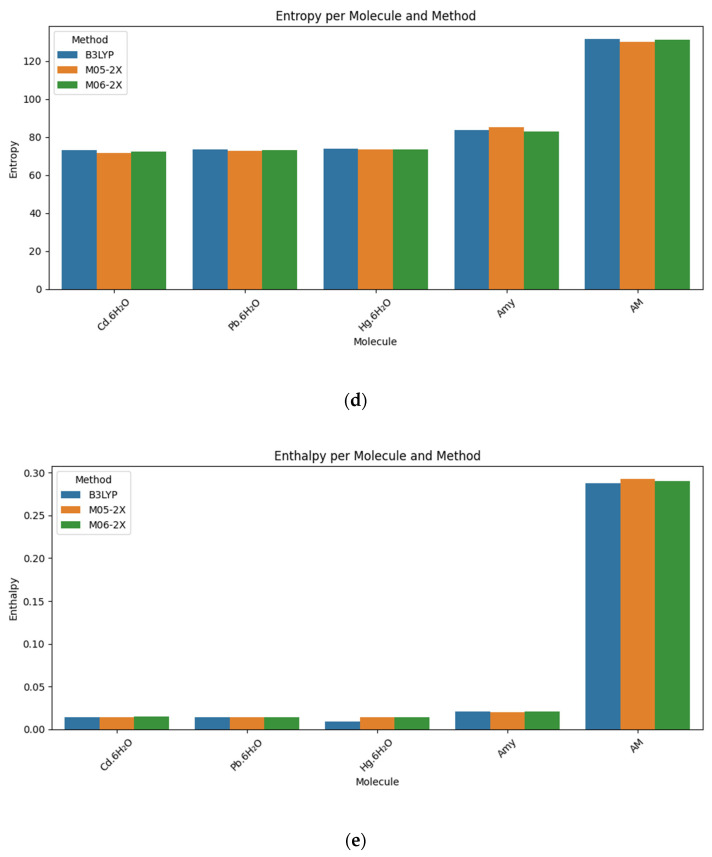
Comparative analysis of energetic and electronic properties of hydrated metal complexes and polymeric fragments using different DFT methods with (**a**) total energy, (**b**) Gibbs free energy, (**c**) total dipole moment, (**d**) entropy, and (**e**) enthalpy.

These trends are visually reinforced in Figure 1, which provides a comprehensive graphical comparison of the total energy, Gibbs free energy, total dipole moment (TDM), entropy, and enthalpy for the hydrated metal complexes (M·6H_2_O) and the individual polymer structures (AM and Amy), as calculated by the B3LYP, M05-2X, and M06-2X methods. This visual representation facilitates a comparative analysis of the thermo-electronic parameters and overall chemical behavior and stability of the modeled polymer fragments.

Figure 1 visually reinforces the aforementioned trends, providing a comprehensive graphical comparison of the thermo-electronic parameters. Figure 1a shows that the B3LYP method consistently predicts the lowest total energies for all studied molecules, indicating superior thermodynamic stability compared to the M05-2X and M06-2X methods. This trend extends to the Gibbs free energy (Figure 1b), where B3LYP generally exhibits more negative values, further reinforcing its capability to describe more stable states under standard conditions. Regarding the total dipole moment (Figure 1c), greater variability is observed between methods, with M05-2X notably predicting the highest TDM values in some metal complexes, which may be attributed to its enhanced representation of polarization and electrostatic interaction potential. Conversely, the entropy values (Figure 1d) display strong consistency across all three methods, suggesting similar vibrational descriptions. While enthalpy (Figure 1e) exhibits slight variations, B3LYP again provides a more uniform balance between internal energy and thermal effects. Through these clear visual interpretations, Figure 1 conclusively demonstrates that the B3LYP/6-311++G(d,p) hybrid functional is a reliable and suitable method for studying molecules with affinity for heavy metals, offering consistent predictions in terms of stability, polarity, and thermoelectric properties.

### 3.2. Model Structures

The model structures for the AM, Amy, and AM/Amy copolymer fragments were specifically designed to simulate their behavior in the presence of hydrated heavy metal ions (M·6H_2_O) and to evaluate their heavy metal removal efficiency. To achieve this, the polymer segments (AM, Amy, and AM/Amy copolymer) were represented as dimers, a size chosen to reduce computational complexity while retaining essential local bonding characteristics.

**Figure 2:**
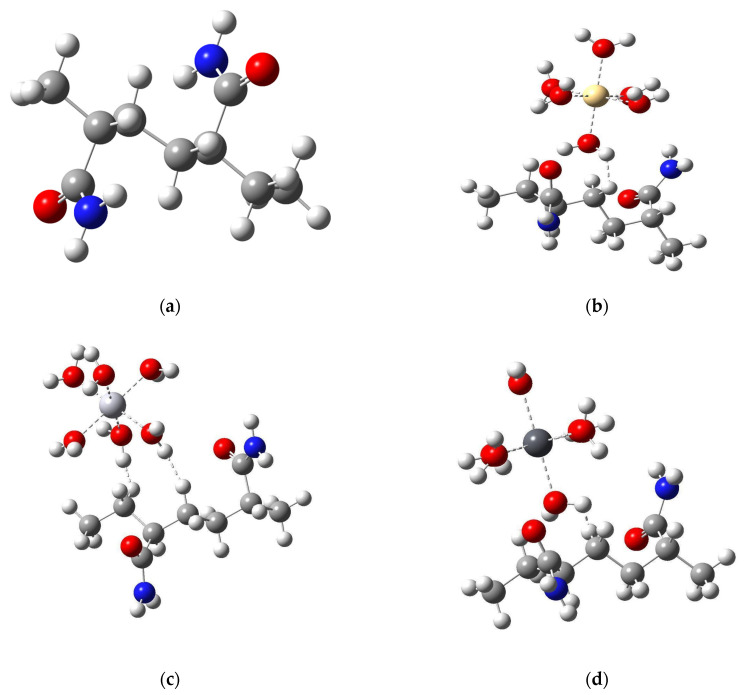

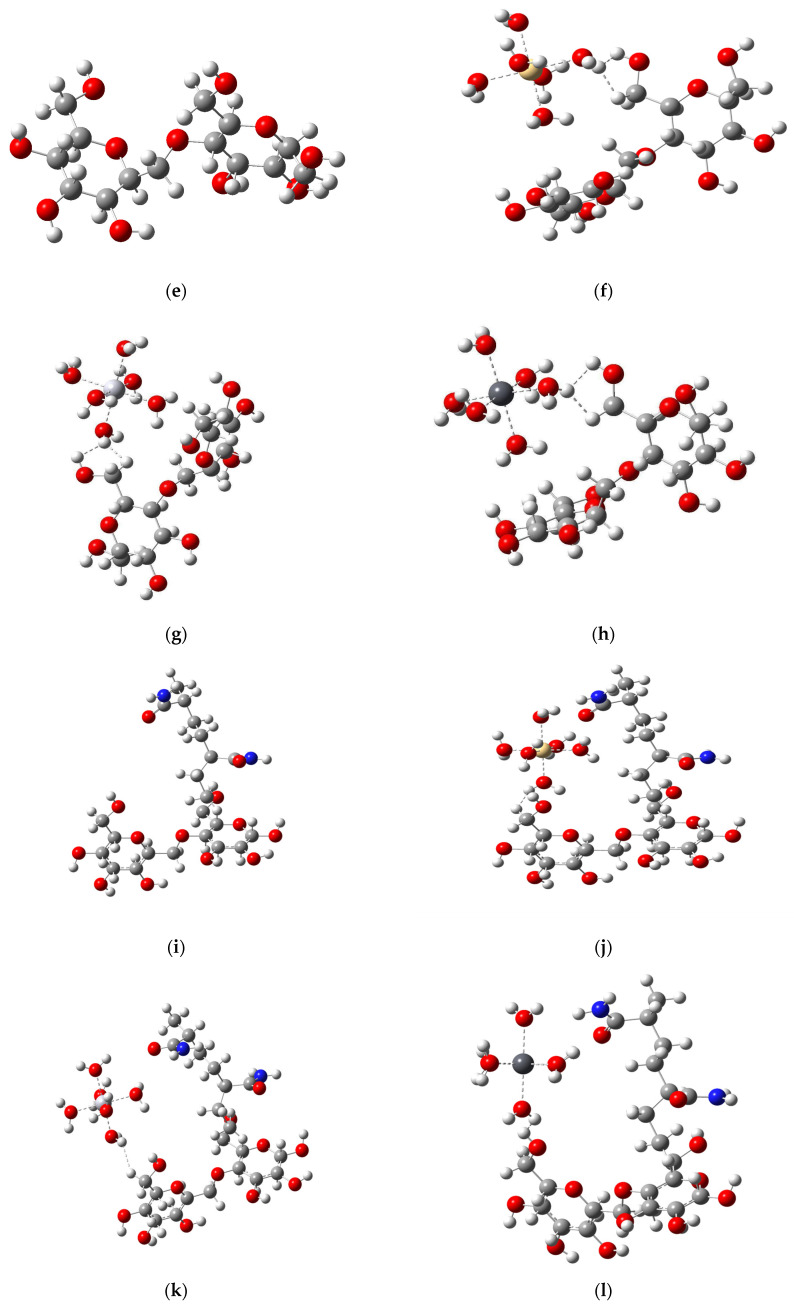
Optimized structures of AM, Amy, and AM/Amy complexes with heavy metals (Cd, Hg, Pb) at the B3LYP/6-311++G(d,p) level of theory: (**a**) AM, (**b**) AM-Cd, (**c**) AM-Hg, (**d**) AM-Pb, (**e**) Amy, (**f**) Amy-Cd, (**g**) Amy-Hg, (**h**) Amy-Pb, (**i**) AM/Amy, (**j**) AM/Amy-Cd, (**k**) AM/Amy-Hg, (**l**) AM/Amy-Pb.

The simulations explicitly considered the direct interaction between the metal ions and the polymer matrices. Depending on the nature of these metal–ligand interactions, the displacement of one or two water molecules from the metal’s coordination sphere was observed. Specifically, when the metal ion established two coordination bonds with the polymer, this consistently involved the displacement of two water molecules. The various structural configurations generated from these interactions are presented in Figure 2.

For a detailed understanding of the reactivity and electronic stability of the modeled dimers of the polymer, density functional theory (DFT) calculations (using the B3LYP hybrid method and the 6-311++G(d,p) basis set) were employed to calculate key electronic parameters. These included molecular electrostatic potential (MEP), total dipole moment (TDM), and frontier molecular orbitals (HOMO–LUMO) along with their energy gap. Such parameters are crucial indicators of a compound’s chemical behavior, polarity, and its propensity for interaction with metallic species.

### 3.3. HOMO-LUMO Calculations

**Figure 3:**
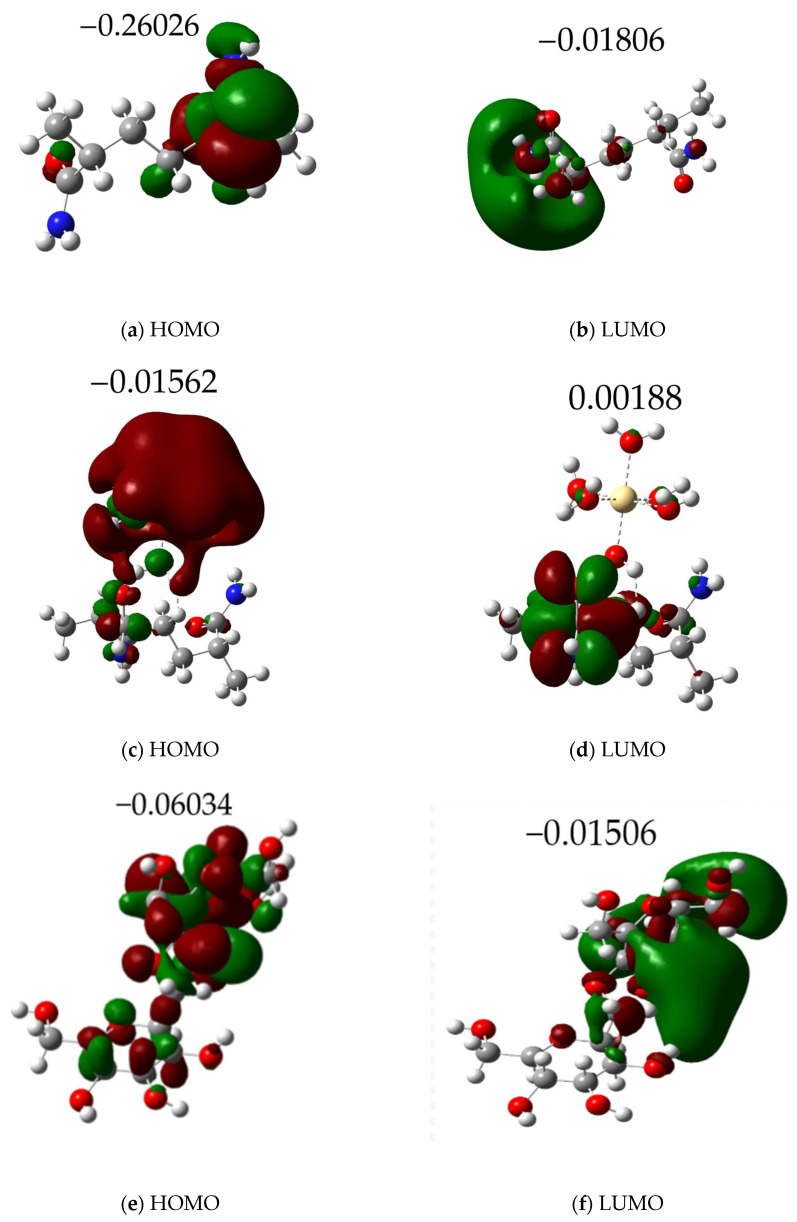

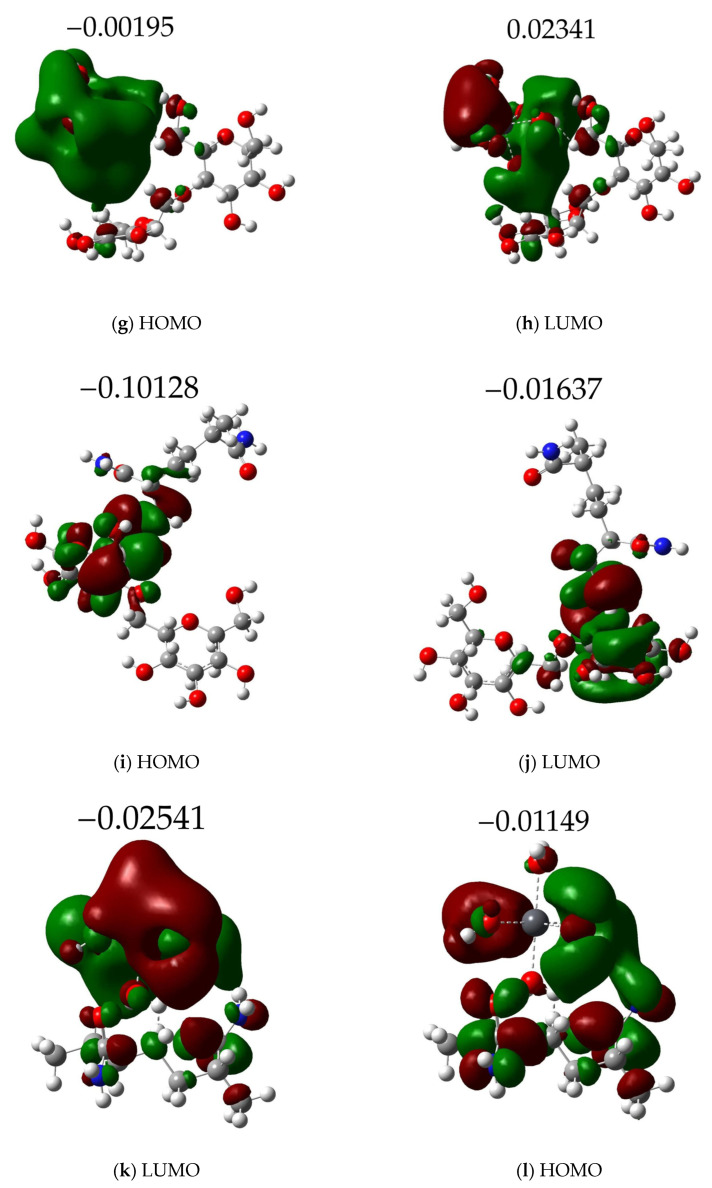
HOMO–LUMO energy levels of optimized structures calculated at the B3LYP/6-311++G(d,p) level: (**a**,**b**) AM, (**c**,**d**) AM-Cd, (**e**,**f**) Amy, (**g**,**h**) Amy-Pb, (**i**,**j**) AM/Amy, (**k**,**l**) AM/Amy-Pb.

To understand the electronic interactions influencing metal adsorption, we calculated the HOMO–LUMO energy gaps and total dipole moments (TDMs) for the complexes formed between acrylamide, amylose, and the acrylamide–amylose copolymer with hydrated Cd, Pb, and Hg metal ions (M·6H_2_O). All calculations were performed under the same computational parameters to ensure a consistent comparison between the molecules. Figure 3 provides visual representations of these frontier molecular orbitals, while Table 2 summarizes their energy values and the calculated TDMs.

Figure 3 illustrates the distribution of the frontier molecular orbitals (HOMO and LUMO) for both the free polymer fragments and their metal complexes.

For the metal-free molecules (Amy, AM, and AM/Amy copolymer), it is observed that the frontier orbitals—both HOMO and LUMO—are distributed relatively uniformly throughout the molecular structure. This indicates that the electron density associated with these orbitals is delocalized and not concentrated in any single specific region.

In stark contrast, when these same polymer molecules interact with heavy metal ions, a significant relocalization of electron density occurs. As clearly shown in Figure 3, both the HOMO and LUMO become highly concentrated around the metallic center. This pronounced redistribution of electron density strongly suggests a robust electronic interaction between the metal and the polymer ligand, evidencing a process of partial charge transfer or electronic coordination. Furthermore, this behavior provides compelling support for the hypothesis of stable complex formation, which is entirely consistent with the negative adsorption energies previously observed.

Table 2 presents the calculated frontier molecular orbital energies (HOMO and LUMO), their energy gap (ΔE), and the total dipole moment (TDM). These values correspond to the complexes formed between the Amy, AM, and the AM/Amy copolymer fragments and the heavy metals Cd, Pb, and Hg.

In the computational simulations of the Amy polymer with metals, Pb exhibited the most favorable values, with the lowest energy gap (ΔE = 0.02613 eV) and the highest dipole moment (TDM = 7.18 Debye). These values indicate a high electronic affinity and effective polarization of the molecules towards this metal. Conversely, Amy complexes with Cd and Hg showed higher ΔE values (0.04528 and 0.04970 eV, respectively) and lower TDMs (<4.5 Debye), indicative of a reduced reactivity and polarization ability compared to those of Pb.

For the AM dimer complexes, the most favorable interaction was observed with Cd, which exhibited the lowest ΔE of the group (0.01750 eV) and a TDM of 5.77 Debye, suggesting high electronic reactivity towards this metal. While AM also interacted with Pb and Hg, their energy gaps were larger (0.02536 and 0.02762 eV, respectively), despite TDM values above 5 Debye. This indicates a comparatively lower effectiveness for Pb and Hg than for Cd.

Significantly, the AM/Amy copolymer segment outperformed both individual polymer segments in all cases. The ΔE values obtained for AM/Amy-Hg (0.01372 eV) and AM/Amy-Pb (0.01392 eV) were the lowest recorded across all molecules, signifying a greater facility for electron transfer. Furthermore, the corresponding dipole moments (11.01 Debye for Hg and 10.79 Debye for Pb) were considerably higher, reflecting an enhanced polarization of the molecules and thus a superior ability to interact with metal cations. Even for Cd, the copolymer showed a significantly reduced ΔE (0.01996 eV) compared to Amy (0.04528 eV). While the ΔE was comparable to that of AM (0.01750 eV), its notably higher TDM (11.01 Debye) further reinforced the copolymer’s overall efficiency for Cd.

These results collectively demonstrate that the AM/Amy copolymer exhibits a synergistic behavior, effectively combining the high electronic reactivity observed in AM with the significant polarization capacity evident in Amy. This translates into an improved adsorption performance across all tested heavy metals, positioning the hybrid copolymer as the most promising for Cd, Hg, and Pb removal in wastewater applications.

The global chemical reactivity descriptors were subsequently calculated from the energies of the frontier orbitals (HOMO and LUMO), obtained via DFT calculations using the B3LYP functional. The equations from conceptual density functional theory used for these calculations are as follows:

(1) $$\mathrm{Ionization}\;\mathrm{potential}\;(I):\;I=-E_{HOMO}$$

(2) $$\mathrm{Electronic}\;\mathrm{affinity}\;(A):\;A=-E_{LUMO}$$

(3) $$\mathrm{Electronegativity}\;(\chi ):\;\chi =\frac{I+A}{2}$$

(4) $$\mathrm{Chemical}\;\mathrm{potential}\;(\mu ):\;\mu =-\frac{I+A}{2}$$

(5) $$\mathrm{Hardness}\;(\eta ):\;\eta =\frac{I-A}{2}$$

(6) $$\mathrm{Softness}\;(S):\;S=\frac{1}{\eta }$$

(7) $$\mathrm{Electrophilicity}\;\mathrm{index}\;(\omega ):\;\omega =\frac{\mu^{2}}{2\eta }$$

where *E_HOMO_* and *E_LUMO_* correspond to the energies of the highest occupied molecular orbital and the lowest unoccupied molecular orbital, respectively, expressed in electron volts (eV). The results of these calculations are presented in Table 3.

In its free state, Amy exhibits moderate softness (15.36 eV) and low electrophilicity (3.47 × 10^−4^ eV), suggesting a limited electron-accepting ability but some potential for electronic adaptability. When complexed with heavy metals, Amy displays metal-dependent behaviors. Amy-Pb, for instance, shows the highest electrophilicity within this ensemble (1.75 × 10^−5^ eV) and a reasonable softness (40.24 eV), indicating a favorable disposition to stabilize the metal charge. In contrast, Amy-Cd and Amy-Hg complexes present higher softness values (44.17 and 76.54 eV, respectively), with Amy-Hg being notably high. However, their very low electrophilicity values (1.61 × 10^−5^ eV for Cd and 3.5 × 10^−6^ eV for Hg) imply that these interactions are primarily influenced by electronic flexibility rather than a strong propensity to attract electron density.

The AM in its free state is characterized by a higher hardness (0.1211 eV) and lower softness (8.26 eV) compared to Amy, alongside a slightly higher electrophilicity (1.17 × 10^−3^ eV). This suggests that AM is less electronically adaptive but possesses a greater inherent tendency to accept electrons. Upon forming complexes with metals, however, AM shows a significant decrease in electrophilicity (2.0 × 10^−7^ eV for AM-Cd, 7.0 × 10^−7^ eV for AM-Hg, and 5.0 × 10^−7^ eV for AM-Pb), while maintaining softnesses between 72 and 114 eV. This behavior reflects that although AM can form relatively flexible complexes, particularly with Cd, its ability to stabilize charge via electron acceptance is limited, potentially affecting its overall metal–ligand interaction efficiency.

Conversely, the free-state AM/Amy copolymer segment stands out for its extraordinary softness (30.57 eV), the highest among the metal-free molecules, indicative of excellent adaptability to electronic stimuli. Its electrophilicity, however, remains the lowest (7.69 × 10^−5^ eV), suggesting a poor inherent ability to accept electron density. Upon the incorporation of heavy metals, this dimer undergoes a dramatic change: its softness reaches maximal values of 100.2 eV for Cd, 143.68 eV for Hg, and 145.77 eV for Pb, representing the highest values across the entire dataset. Although the electrophilicity of these complexes remains low (on the order of 10^−6^ eV), the extreme softness of the AM/Amy copolymer fragment enables it to efficiently reorganize in the presence of metallic charges, thereby favoring the formation of highly stable complexes. This behavior is particularly prominent in the cases of AM/Amy-Pb and AM/Amy-Hg, where the exceptional electronic flexibility compensates for the limited intrinsic electron-accepting tendency, facilitating highly efficient interactions.

Comparing Amy, AM, and AM/Amy reveals a crucial contrast between electronic acceptance capacity (ω) and adaptability (S), which fundamentally dictates how each molecule interacts with specific metal ions. This intricate balance between rigidity, reactivity, and flexibility defines the differential response of each polymer fragment to Cd, Hg, and Pb metals.

These insights from the global reactivity descriptors, particularly the dramatically increased softness (S) values and correspondingly decreased hardness (η) upon metal complexation, are critical for understanding the nature of metal–polymer interactions. For all the investigated molecules, the formation of complexes with heavy metals leads to a significant increase in softness compared to the free polymers (e.g., the softness for AM/Amy rises from 30.57 eV to over 100 eV for metal complexes, reaching up to 145.77 eV for AM/Amy-Pb). This substantial enhancement in softness, coupled with the observed concentration of frontier molecular orbital density around the metal centers (as shown in Figure 3), serves as compelling evidence for a chemisorptive mechanism. This strong chemical interaction is crucial for the high adsorption efficiencies observed for these polymer fragments in wastewater treatment.

### 3.4. DOS Analysis

**Figure 4:**
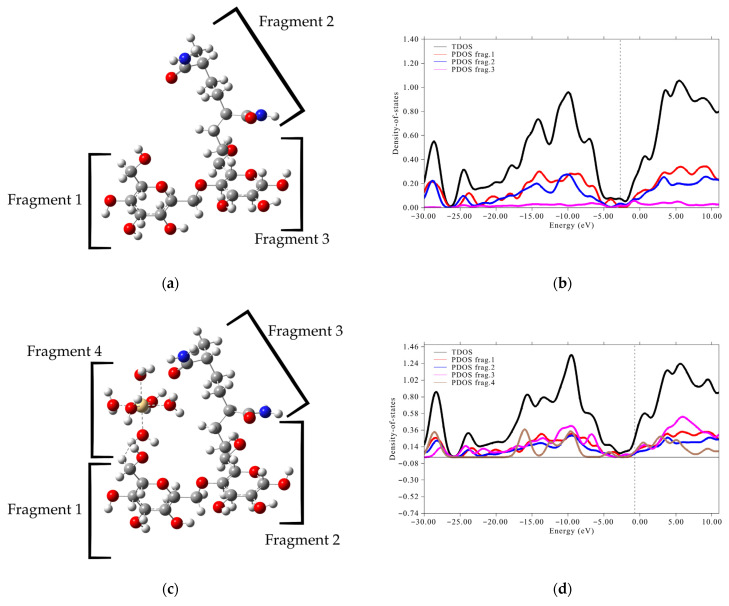

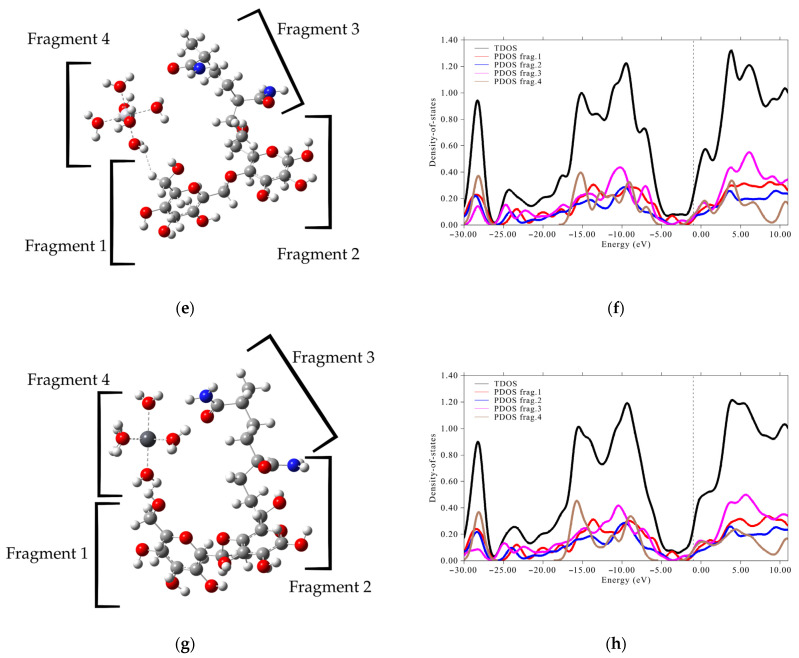
DOS y PDOS graphs calculated at the B3LYP/6-311++G(d,p) level: (**a**,**b**) AM/Amy, (**c**,**d**) AM/Amy-Cd, (**e**,**f**) AM/Amy-Hg, (**g**,**h**) AM/Amy-Pb.

To further elucidate the electronic properties and interactions discussed, we performed Density of States (DOS) analyses. These analyses offer a detailed picture of how electronic states are distributed across different energy levels within the polymer systems and their hydrated metal by examining the contributions of individual fragments, namely, the amylose and acrylamide segments, as well as the complexed metal ions (Cd, Hg, Pb). The DOS graphs reveal their specific roles in the overall electronic structure and reactivity. This allows us to precisely identify which components are involved in electron transfer processes and how their electronic overlap near the Fermi level dictates the stability and nature of the metal–polymer interactions as shown in Figure 4.

The comprehensive analysis of the Density of States (DOS) graphs for the amylose (Amy) and acrylamide (AM) molecules, as well as their complexes with hydrated Cd, Hg, and Pb ions, provides profound insights into their electronic contributions and interaction mechanisms. We consistently observe that amylose (Amy) fragments play a predominant role in the molecule’s electronic reactivity, showing significant participation in the frontier orbital region near the Fermi level (0 eV). This positions them as the primary sites for interaction and electron transfer with the metals. In contrast, the acrylamide (AM) dimer exhibits its density of states primarily at deeper energy levels, suggesting a more structural function and less direct influence on the chemical reactivity of the frontier orbitals.

Crucially, in all the studied complexes (Cd·6H_2_O, Hg·6H_2_O, and Pb·6H_2_O), a clear localization of the metal ions’ electronic density is observed in the proximity of the Fermi level. This electronic overlap is a robust indicator of strong interaction and charge redistribution between the metal ion and the organic polymer fragments, particularly amylose. This evidence strongly supports the formation of stable adsorption complexes and suggests an effective interaction mechanism, based on the polymers’ ability to accommodate and stabilize the metal charge through electronic reconfiguration. In essence, the electronic properties of amylose are key for heavy metal capture, with acrylamide contributing to the material’s overall stability.

### 3.5. Molecular Electrostatic Potential

The molecular electrostatic potential (MEP) map is a graphical representation that illustrates the charge distribution within a molecule, reflecting how the electronic environment is shaped by atomic nuclei and electrons. This map is typically visualized using a chromatic scale: red regions indicate areas of high negative electrostatic potential, corresponding to a high electron density (nucleophilic sites), while blue regions represent areas of high positive potential, characterized by low electron density (electrophilic sites). This visual tool is highly beneficial for predicting potential electrostatic interaction sites between molecules, aiding in the identification of electron donor or acceptor regions.

**Figure 5:**
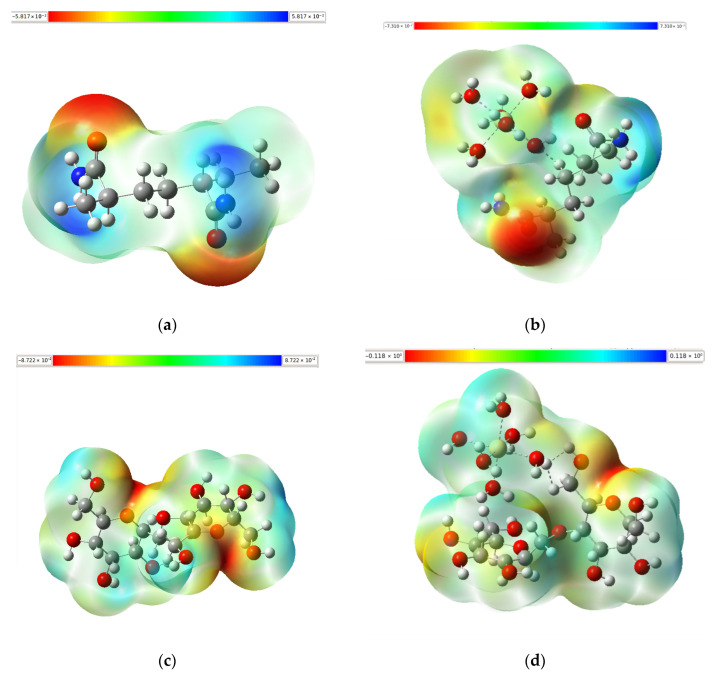

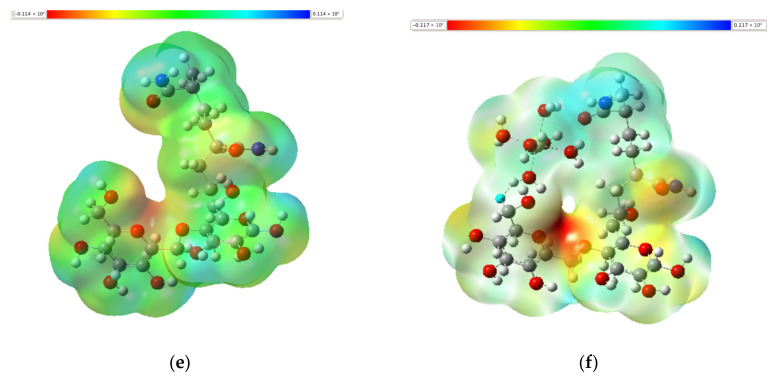
Molecular electrostatic potential (MEP) maps of optimized structures calculated using B3LYP/6-311++G(d,p) for (**a**) AM, (**b**) AM–metal, (**c**) Amy, (**d**) Amy–metal, (**e**) AM/Amy copolymer, and (**f**) AM/Amy–metal.

As depicted in Figure 5, the MEP maps of the studied polymer fragments (AM, Amy, and AM/Amy copolymer) clearly highlight areas of significant electron accumulation, predominantly shown in red. These electron-rich regions are primarily localized around oxygen atoms, which are integral to various functional groups such as hydroxyls (-OH) and ethers (-O-) present in the polymer structures, including the furanose-type heterocyclic rings characteristic of amylose. This distinct distribution indicates that these oxygen atoms serve as primary reactivity centers in the molecules. Their high electron density facilitates the strong electrostatic attraction and subsequent adsorption of heavy metal ions, enabling the formation of direct coordination bonds with the metals.

Regarding the absorption mechanism, the highly negative electrostatic potential (red regions) around these oxygen atoms signifies that they are rich in electron density, making them excellent Lewis basic sites. Positively charged heavy metal ions, acting as Lewis acids, are strongly attracted to these electron-dense regions via electrostatic forces. Upon close approach, the lone-pair electrons from these oxygen atoms are donated to the metal ions, forming strong direct coordination bonds. This process inherently involves the displacement of water molecules from the metal’s primary hydration sphere, leading to the effective immobilization of the metal ion onto the polymer surface.

## 4. Conclusions

The computational analysis employing density functional theory (DFT) provided profound insights into the distinct interaction capabilities of the Amy, AM, and AM/Amy polymeric fragments with heavy metal ions (Cd, Hg, and Pb). The Amy polymer demonstrated an intermediate interaction profile. Its energy gaps (ΔE), derived from the separation of its frontier molecular orbitals as visualized in the Density of States (DOS) graphs, ranged from 0.02613 to 0.04970 eV, with moderate dipole moments, suggesting an acceptable reactivity, particularly towards Pb. For Amy-Pb, the lowest ΔE (0.02613 eV) and highest TDM (7.18 Debye) within the Amy complexes were observed, indicating its most favorable interaction. In contrast, the AM polymer exhibited higher electronic reactivity specifically towards Cd (ΔE = 0.01750 eV, TDM = 5.77 Debye). However, its overall electronic adaptability was comparatively lower, as indicated by its higher hardness (0.1211 eV) and reduced softness (8.26 eV), with its electronic states primarily localized at deeper energy levels in the DOS profile, suggesting a more structural role.

Significantly, the AM/Amy copolymer demonstrated a synergistic integration of the individual advantages of Amy and AM, resulting in superior performance. It consistently showed the lowest energy gaps across the entire dataset, excelling notably with Hg (0.01372 eV) and Pb (0.01392 eV). Concurrently, it displayed the highest dipole moments (TDM > 10 Debye), indicative of exceptional polarization and electronic interaction capability. This remarkable performance was further complemented by the highest observed softnesses (S > 100 eV), signifying outstanding electronic flexibility and adaptability in response to external metallic stimuli. While all the polymer fragments generally exhibited low electrophilicities (ω in the order of 10^−6^ eV), the AM/Amy copolymer effectively compensated for this limitation. Its exceptionally high softness, further corroborated by the significant electronic contributions near the Fermi level observed in its DOS graphs, facilitated the efficient electronic reorganization necessary for the stable complexation of heavy metals. Furthermore, molecular electrostatic potential (MEP) maps consistently confirmed that the primary reactive sites across all molecules are indeed the oxygen atoms. Critically, these maps revealed a greater electron accumulation in the copolymer, reinforcing its enhanced nucleophilicity compared to that of the individual polymers.

The AM/Amy copolymer offers an optimal balance between electronic reactivity and flexibility, consistently outperforming both Amy and AM across key indicators of metal–ligand interaction. Its unique synergistic behavior positions it as the most efficient polymer fragment identified in this study for removing heavy metals from aqueous solutions, making it an ideal candidate for environmental applications in the decontamination of water loaded with cations such as Cd, Hg, and Pb.

## Figures and Tables

**Table 1:** Energetic and dipolar properties of metal–water complexes and copolymer segments using different DFT methods.

Molecule	Method/Base Set	Energy (Hartree)	Gibbs Free Energy (Hartree)	TDM (Debye)	Entropy (Cal/Mol-Kelvin)	Enthalpy (Hartree)
Cd·6H_2_O	B3LYP/6-311++G(d,p)	−506.402385	−0.020369	1.7821	73.214	0.014417
	M05-2X/6-31++G(d,p)	−506.122292	−0.020156	2.8165	71.839	0.013977
	M06-2X/6-311+G(d,p)	−506.094871	−0.019112	2.3847	72.427	0.015300
Pb·6H_2_O	B3LYP/6-311++G(d,p)	−500.978698	−0.020813	0.6991	73.486	0.014102
	M05-2X/6-31++G(d,p)	−500.706202	−0.020537	0.6189	72.850	0.014077
	M06-2X/6-311+G(d,p)	−500.660737	−0.020567	0.6241	73.257	0.01424
Hg·6H_2_O	B3LYP/6-311++G(d,p)	−461.812930	−0.025824	1.1458	73.841	0.009260
	M05-2X/6-31++G(d,p)	−461.596211	−0.021032	2.0222	73.433	0.013858
	M06-2X/6-311+G(d,p)	−461.494978	−0.020732	1.6838	73.462	0.014172
Amy	B3LYP/6-311++G(d,p)	−1376.086739	−0.019303	2.5127	83.814	0.02052
	M05-2X/6-31+G(d,p)	−1376.481552	−0.020062	2.5264	85.329	0.020480
	M06-2X/6-311++G(d,p)	−1375.525214	−0.018545	1.7550	83.108	0.020942
AM	B3LYP/6-311++G(d,p)	−613.991554	0.224943	4.1389	131.765	0.287548
	M05-2X/6-31+G(d,p)	−613.760652	0.230967	4.2997	130.003	0.292735
	M06-2X/6-311++G(d,p)	−613.714295	0.228123	4.1722	131.258	0.290488

**Table 2:** Frontier molecular orbital energies (HOMO, LUMO), energy gap (ΔE), and dipole moment (TDM) of copolymer–metal complexes in aqueous phase.

Molecule	Metal	Lumo (eV)	Homo (eV)	ΔE (eV)	TDM (Debye)
Amy	Cd·6H_2_O	−0.01506	−0.06034	0.04528	4.32257
	Hg·6H_2_O	−0.01267	−0.06237	0.04970	4.08872
	Pb·6H_2_O	−0.01018	−0.03631	0.02613	7.17751
AM	Cd·6H_2_O	0.00188	−0.01562	0.01750	5.77141
	Hg·6H_2_O	0.00503	−0.02259	0.02762	5.41831
	Pb·6H_2_O	0.02341	−0.00195	0.02536	5.82425
AM/Amy	Cd·6H_2_O	−0.01637	−0.03633	0.01996	11.00673
	Hg·6H_2_O	−0.01169	−0.02541	0.01372	8.76457
	Pb·6H_2_O	−0.01149	−0.02541	0.01392	10.79232

**Table 3:** Global reactivity descriptors for Amy, AM, and AM/Amy complexes with heavy metals (Cd, Hg, Pb).

Molecule	Metal	Chemical Potential (μ)	Ionization Potential (I)	Electronegativity (χ)	Electronic Affinity (A)	Electrophilicity (ω)	Hardness (η)	Softness (S)
Amy	-	−0.10320	0.16829	0.10320	0.03811	0.0003466	0.06509	15.3633
AM	-	−0.13916	0.26026	0.13916	0.01806	0.0011726	0.1211	8.2576
AM/Amy	-	−0.06857	0.10128	0.06857	0.03586	0.0000769	0.03271	30.5717
Amy	Cd·6H_2_O	−0.03770	0.06034	0.03770	0.01506	0.0000161	0.02264	44.1696
	Hg·6H_2_O	−0.02325	0.03631	0.02325	0.01018	0.0000035	0.013065	76.5404
	Pb·6H_2_O	−0.03752	0.06237	0.03752	0.01267	0.0000175	0.02485	40.2414
AM	Cd·6H_2_O	−0.00687	0.01562	0.00687	−0.00188	0.0000002	0.00875	114.2857
	Hg·6H_2_O	0.01073	0.00195	−0.01073	−0.02341	0.0000007	0.01268	78.8644
	Pb·6H_2_O	−0.00878	0.02259	0.00878	−0.00503	0.0000005	0.01381	72.4113
AM/Amy	Cd·6H_2_O	−0.02635	0.03633	0.02635	0.01637	0.0000035	0.00998	100.2004
	Hg·6H_2_O	−0.01845	0.02541	0.01845	0.01149	0.0000012	0.00696	143.6782
	Pb·6H_2_O	−0.01855	0.02541	0.01855	0.01169	0.0000012	0.00686	145.7726

## Data Availability

The original contributions presented in this study are included in the article. Further inquiries can be directed to the corresponding author.

## Abbreviations

The following abbreviations are used in this manuscript:

AM	Acrylamide
Amy	Amilose
AM/Amy	Acrylamide-Amilose copolymer
DFT	Density Functional Theory
DOS	Destiny of States
HOMO	Highest Occupied Molecular Orbital
LUMO	Lowest Unoccupied Molecular Orbital
TDM	Total Dipole Moment
MEP	Molecular Electrostatic Potential
